# Effective venetoclax treatment for indolent T-cell lymphoma of the gastrointestinal tract: a case report and literature review

**DOI:** 10.3389/fimmu.2025.1593343

**Published:** 2025-07-01

**Authors:** Ji-Mo Jian, Hong-Yuan Hao, Cheng-Lu Yuan, Shu-Qi Zhang, Zou-Fang Huang, Jun Du

**Affiliations:** ^1^ Department of Hematology, Qilu Hospital of Shandong University, Qingdao, Shandong, China; ^2^ Department of Hematology, Qilu Hospital of Shandong University, Jinan, Shandong, China; ^3^ Department of Hematology, Shanghai Jiao Tong University School of Medicine, Shanghai, China; ^4^ Ganzhou Key Laboratory of Hematology, Department of Hematology, The First Affiliated Hospital of Gannan Medical University, Ganzhou, Jiangxi, China; ^5^ Department of Hematology, Renji Hospital, School of Medicine, Shanghai Jiao Tong University, Shanghai, China; ^6^ Department of Hematology, Punan Hospital, Shanghai, China

**Keywords:** indolent T-cell lymphoma, gastrointestinal tract, hematochezia, venetoclax, B-cell lymphoma 2

## Abstract

Indolent T-cell lymphoma of the gastrointestinal tract (iTCL-GI) is rare, lacking standardized treatments. We report a successful venetoclax treatment in one patient with indolent T-cell lymphoma of the gastrointestinal tract. A 35-year-old male was admitted due to complaints of anemia and hematochezia. He was diagnosed with iTCL-GI according to histopathology and next-generation sequencing (NGS). He received the first cycle of CHOP-E chemotherapy, but he continued to have intermittent blood in stools. After starting oral Bcl-2 inhibitor venetoclax, the results of peripheral hemogram and the body temperature gradually turned normal, with no symptoms of hematochezia occurring again. In addition, colonoscopy showed improved ulcers in the ascending and transverse colon. Routine blood tests returned to normal without adverse effects. Therefore, venetoclax may represent a potential treatment approach for iTCL-GI. This report might provide clues for the future management of similar cases.

## Introduction

Indolent T-cell lymphoma of the gastrointestinal tract (iTCL-GI) is an exceedingly rare monoclonal T-cell proliferative disorder composed of small, mature lymphocytes, primarily affecting the gastrointestinal tract, particularly the small intestine and colon (1). Primary gastrointestinal lymphomas account for only 1%-4% of all gastrointestinal malignancies, with indolent T-cell lymphoma being even rarer—fewer than 40 cases have been reported globally (2, 3). Due to its extremely low incidence, systematic and standardized research on pathological characteristics, pathogenesis, and effective treatments for iTCL-GI has been challenging. Patients with iTCL-GI typically present with symptoms such as diarrhea, weight loss, and abdominal pain. These symptoms overlap significantly with those of more common gastrointestinal disorders, posing substantial challenges to early diagnosis (4, 5). Moreover, the clinical progression of this disease is generally indolent. Despite the favorable prognosis for most cases, persistent symptoms can lead to chronic malnutrition, severely impairing patients’ quality of life (6). Therefore, timely and effective treatment is crucial for improving patient outcomes.

Currently, the treatment of iTCL-GI lacks a standardized protocol and primarily relies on individualized and empirical approaches. Conventional treatment strategies include chemotherapy and immunomodulatory agents (7). However, given that iTCL-GI is a low-grade lymphoma, traditional chemotherapy regimens have demonstrated limited efficacy in some patients and are often associated with significant toxicity (8). Thus, the primary challenge in treating this disease lies in effectively managing disease progression, alleviating symptoms, and minimizing adverse effects. In recent years, venetoclax, a B-cell lymphoma 2 (Bcl-2) inhibitor, has shown promising potential in treating hematologic malignancies (9, 10). Bcl-2 is an anti-apoptotic protein that is overexpressed in various lymphomas, and venetoclax promotes apoptosis of tumor cells by inhibiting Bcl-2, thereby exerting an anti-tumor effect. Notably, venetoclax has demonstrated significant efficacy in treating chronic lymphocytic leukemia (CLL) and certain T-cell lymphomas (11, 12). However, its application in iTCL-GI remains in an exploratory phase, with limited reports in the literature. In this case report, we present a detailed account of a patient with iTCL-GI successfully treated with venetoclax, providing a comprehensive discussion from clinical presentation and diagnostic process to therapeutic outcome. This report aims to assess the feasibility and safety of this therapeutic approach, offering valuable insights for clinicians managing similar cases.

## Case report

On April 27, 2022, a 35-year-old male patient was admitted to the hospital with complaints of anemia, hematochezia, joint pain and morning stiffness (relieved after activity). Elevated levels of rheumatoid factor, erythrocyte sedimentation rate (ESR), and C-reactive protein (CRP) were detected. Ulcers were observed in the oral palate and pharyngeal mucosa, some extending to the gums, without bleeding.

The patient had a history of oral ulcers lasting seven years and experienced knee pain for two months. Since February 2015, he has suffered from severe oral ulcers, pain, general weakness, and fever. An oral antibiotic, minocycline-tetracycline (specific details unknown), was administered at a local hospital, but the ulcers continued to worsen. A biopsy of the ulcers revealed chronic inflammation. The patient was then prescribed prednisone and thalidomide, but there was no improvement in the ulcers. In April 2015, a follow-up biopsy at the local hospital showed chronic inflammation with lymphoid hyperplasia, local necrosis, and infiltration of lymphocytes, leading to consideration of an atypical T-cell lymphoproliferative disorder (TLPD). The patient was treated with methotrexate and folic acid. However, by June 2016, he stopped the medication, citing ineffectiveness. Despite ongoing ulcers and pain, no further treatments were pursued.

**Figure 1:**
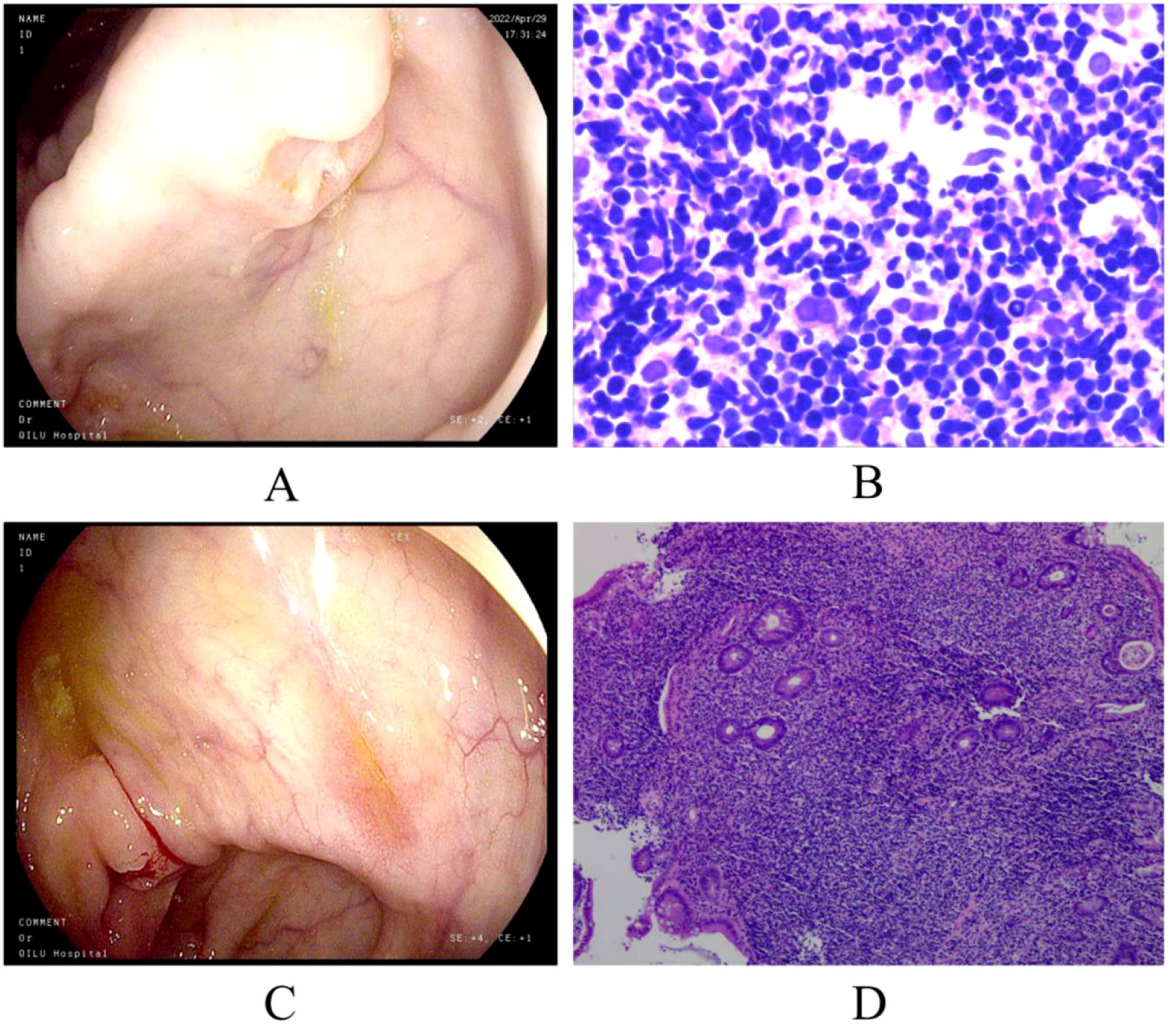
Colonoscopy and pathological biopsy. **(A)** Ceroscopy on April 29, 2022: multiple ulcers of ascending and transverse colon; **(B)** pathological biopsy on 29 April 2022; **(C)** ascending colonoscope on September 28,2022: improvement in the ulcers of the ascending and transverse colon; **(D)** pathological biopsy on 28 September 2022. (We have submitted all the original Files to the NGDC database. Project ID: PRJCA017285.).

On April 28, 2022, routine tests were abnormal and the results showed that White Blood Cell count (WBC) count was 16.04×10^9/L (reference range, 4.0-10.0×10^9/L), Neutrophil count (NEU) count was 9.77×10^9/L (reference range, 2.0-7.5×10^9/L), Hemoglobin (HB) level was 96g/L (reference range, 130–175 g/L), Platelet count (PLT) count was 822×10^9/L (reference range, 150-400×10^9/L), and ESR was 110 mm/h (reference range, 0–15 mm/h). The whole-body CT discovered bilateral pulmonary nodules and gallbladder stones. On April 29, 2022, the patient underwent a gastro-colonoscopy, and multiple ulcers can be seen in the enteric canal. Histopathology revealed T cell proliferative disorders in the cecum, ileocecal junction, ascending colon, transverse colon, descending colon, and rectum (**Figures 1A, B**). Given the patient’s elevated ESR, bone marrow puncture and biopsy were performed. HE and PAS staining revealed no abnormal plasma cells in the bone marrow tissue. Bone marrow aspiration confirmed the involvement of bone marrow. Bone marrow biopsy revealed normal bone marrow hyperplasia, with scattered or small clusters of lymphocytes; some lymphocytes exhibited irregular morphology. Flow cytometry demonstrated a population of abnormal T cells, accounting for 82.35% of lymphocytes (30.72% of nucleated cells). Immunohistochemical analysis revealed the following profile: CD3+, CD4-, CD8+, CD2+, CD5dim, CD7+, CD57-, CD56-, Granzyme B-, Perforin-. The positive expression rate of TRBC1 was 1.48% (biological reference range, 15%-85%). In combination with clinical characteristics, morphological characteristics, and immunohistochemistry findings, a diagnosis of iTCL-GI was considered. During the endoscopic examination, multiple isolated or fused polypoid elevations were observed in the antrum and body of the stomach, with a smooth surface. Light microscopy of the endoscopic submucosal dissection (ESD) samples from the gastric antrum and body revealed diffuse and dense lymphocyte infiltration in the lamina propria, with focal infiltration. The lymphocytes displayed small to medium size with mild nuclear atypia and exhibit a monomorphic appearance. Immunohistochemical analysis showed positive results for CD2, CD3, CD5, CD7, CD8, TIA, and Ki-67 (approximately 5%), indicating the presence of CD8-positive cytotoxic T cells and low proliferative activity of the tumor. However, CD4 and GrB were negative, and small RNA encoded by Epstein-Barr virus (EBV) (EBER) was also negative as determined by *in situ* hybridization. A negative EBER result can help exclude extranodal NK/T-cell lymphoma and EBV-positive mucocutaneous ulcer (EBVMCU). The supplementary immunohistochemistry report indicated Bcl-2 positivity. Additionally, using PCR amplification technique, gene rearrangement tests detected clonal rearrangement of the T lymphocyte receptor genes TCRγ (TCRG) and TCRβ (TCRB) were positive. We conducted next-generation sequencing (NGS), systematically sequencing approximately 22,000 genes. The NGS identified several mutations, including DDX3X exon 7 c. 659T>C p.L220S, DDX3X exon 7 c. 560T>G p.M187R, and JAK2 exon 20 c. 2696T>C p.I1889T. The mutation frequencies were found to be 3.1% for DDX3X p.L220S, 56.9% for DDX3X p.M187R, and 48.9% for JAK2 p.I1889T. Ultimately, the patient was diagnosed with iTCL-GI based on histopathology and TCR gene rearrangement.

The patient received a range of symptomatic treatments, including anti-infection therapy, acid suppression treatment, nutritional support, blood component transfusions, albumin supplementation, and hemostatic treatment. In mid-June 2022, he received a first cycle of CHOP-E chemotherapy, which included cyclophosphamide (750 mg/m²), vincristine (4 mg), and doxorubicin (70 mg/m²) on day 1, etoposide (100 mg/m²) on days 1-3, and prednisolone (80 mg) on days 1-5. During chemotherapy, the patient developed a fever due to myelosuppression and was treated with meropenem in combination with linezolid. He also received leucocyte-enhancing agents to boost his leucocyte levels, along with additional blood component transfusions and nutritional support. He was discharged after his condition improved, but he continued to have intermittent bloody stools at home. Due to poor tolerance of and lack of response to CHOP-E chemotherapy, coupled with positive Bcl-2 immunohistochemical results, he commenced oral venetoclax therapy combined with allopurinol in mid-July. Rapid tumor shrinkage may occur during treatment, so prophylactic hydration and uric-lowering medications can be administered before the first dose to reduce the risk of tumor lysis syndrome.The venetoclax dosage followed the standard schedule used in CLL: 20 mg/day in week 1, 50 mg/day in week 2, 100 mg/day in week 3, 200 mg/day in week 4, and 400 mg/day from week 5. Throughout the entire venetoclax treatment process, the patient tolerated therapy well without requiring any dose modifications.

**Figure 2:**
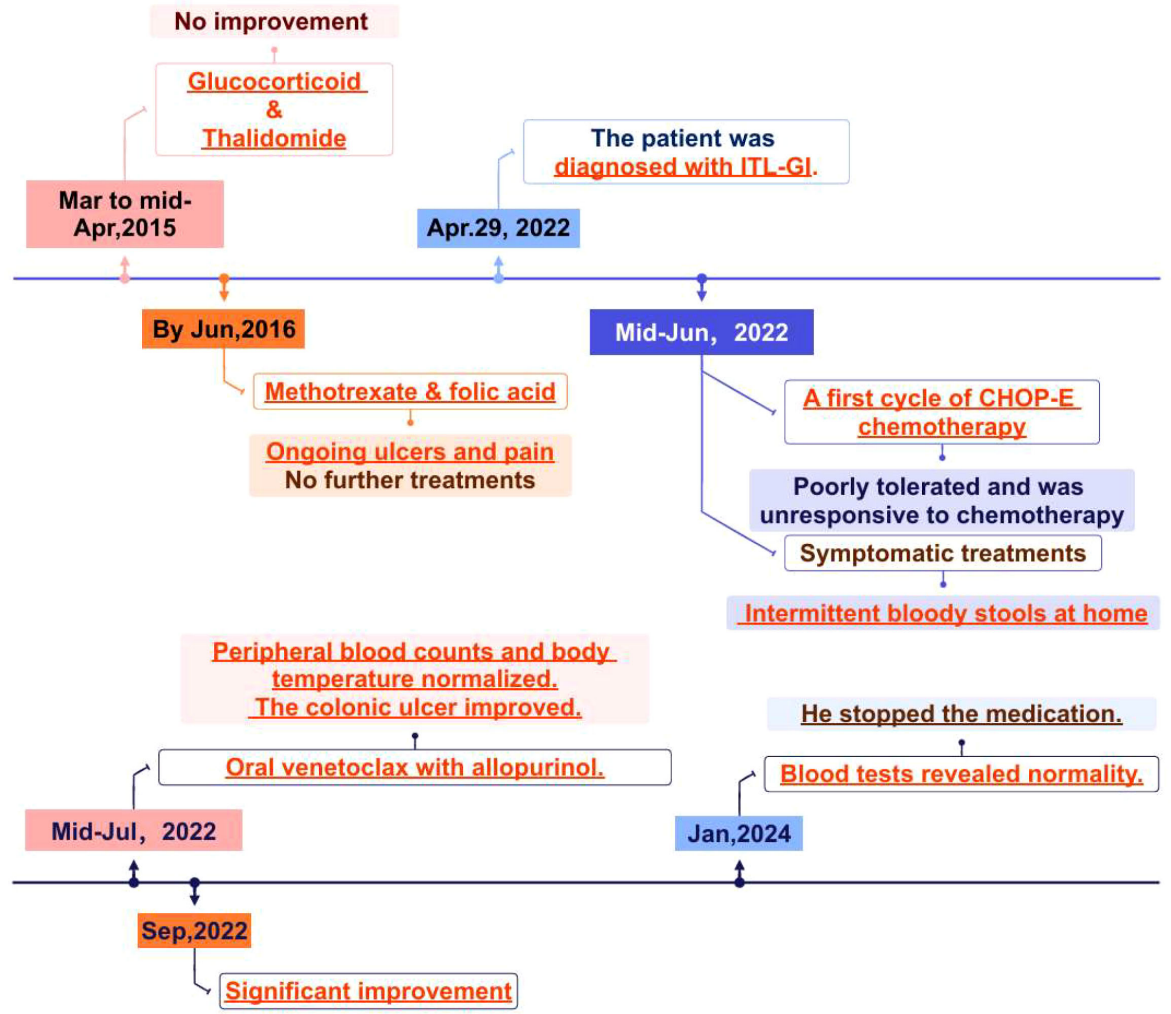
Diagram of treatment history.

After treatment with venetoclax, the patient’s blood counts and temperature gradually returned to normal, and there was no recurrence of blood in the stool. The colonoscopy showed improvement in the ulcers of the ascending and transverse colon (**Figure 1C**). In late September, the pathological biopsy indicated significant improvement (**Figure 1D**). The patient’s general condition recovered and is still under close follow-up. On January 09, 2025, the latest routine blood tests revealed the WBC count of 7.83×10^9/L, the NEU count of 4.00×10^9/L, the LYM count of 3.23×10^9/L, the Monocyte count of 0.52×10^9/L, the HB level of 144g/L, and the PLT count of 276×10^9/L. Following completion of treatment, the patient underwent bone marrow reevaluation, and flow cytometry demonstrated no minimal residual disease (MRD). As of now, it has been 30 months since treatment and no hematological toxicity and hepatorenal dysfunction were observed. **Figure 2** provides a comprehensive overview of the patient’s treatment trajectory. For the treatment of iTCL-GI, considering the risk of venetoclax inducing secondary tumors (13), we need to be cautious in our clinical treatment strategies. During the initial diagnosis and treatment stage, combination that may induce secondary tumors should be avoided. Long term use of venetoclax requires close monitoring, especially in combination drug strategies. In this case, the patient has stopped using venetoclax after achieving complete remission of the disease, and no secondary tumors have occurred. The patient remain in good health and is still under close follow-up.

## Discussion

A 35-year-old male diagnosed with iTCL-GI received CHOP-E chemotherapy, however reached no improvements. Since July 2022, the patient has been treated with venetoclax over a cumulative treatment period of 30 months. During this period, the patient’s routine blood tests returned to normal, and his body temperature was maintained within the normal range, while the ulcers in the ascending and transverse colon improved significantly. Suspension of the drug and observation may now be considered. Therefore, venetoclax shows potential as a viable therapeutic option for the treatment of iTCL-GI. This case report provides an important reference point and clinical insight for the management of similar cases in the future.

iTCL-GI is a rare T-cell-derived non-Hodgkin’s lymphoma that is typically characterized as a low-grade malignant lymphoma, accounting for approximately 1%-4% of all gastrointestinal malignancies (14). The disease was first reported in 2013 by Perry AM et al., who described 10 patients with small-cell clonal T-cell lymphoproliferations involving the lamina propria of the gastrointestinal tract (15). iTCL-GI primarily affects middle-aged males, with common clinical manifestations including chronic diarrhea, weight loss, and abdominal pain (16). Owing to its nonspecific clinical features, it is often confused with inflammatory bowel disease, irritable bowel syndrome, and other gastrointestinal tumors. The disease may involve the entire gastrointestinal tract, most commonly affecting the small intestine and colon, followed by the oral cavity, stomach, esophagus, and occasionally the bone marrow and peripheral blood (17). The clinical presentation of this patient included chronic oral ulcers, joint pain, fever, and blood in the stool. Initial biopsy revealed chronic inflammation with lymphoid hyperplasia, leading to a preliminary diagnosis of atypical T-lymphoproliferative disease, however, treatment resulted in no significant improvement. There is a lack of specific diagnostic markers for iTCL-GI, and common endoscopic manifestations include mucosal nodules, small polyps, and superficial ulcers (16). However, in some cases, there may be no obvious abnormality during routine examinations, and ultimately, the diagnosis is confirmed by biopsy. The pathomorphological manifestations of iTCL-GI include small lymphoid cells located in the lamina propria in a diffuse distribution without epitheliotropism (18). The pathomorphological features of our patient were consistent with these characteristics.

Immunophenotypic testing is an important diagnostic tool, and the lymphoid cells in iTCL-GI demonstrate a mature T-cell phenotype, with the majority being CD4+/CD8-, and to a lesser extent CD4-/CD8+ (14, 19). In our patient, the cells showed a CD4-/CD8+ and CD56-negative phenotype, indicating that the tumor cells were predominantly cytotoxic T-cells (CD8+ T-cells), which are involved in mediating immune responses and killing tumor cells. In diagnosing iTCL-GI, TCR gene rearrangement and NGS are valuable tools for determining its genetic characteristics. TCR gene rearrangement is a typical molecular hallmark of T-cell lymphomas, indicating that the tumor cells undergo clonal proliferation (20). In this case, the TCR gene rearrangement test was positive, consistent with iTCL-GI diagnostic criteria, suggesting the presence of monoclonal T-cell proliferation. Furthermore, NGS testing identified mutations in the JAK2 and DDX3X genes in this patient, indicating that the disease is heterogeneous at the genetic level and may contribute to the malignant transformation of tumor cells (21). It has been hypothesized that genetic alterations in the JAK-STAT signaling pathway, as well as mutations in genes encoding epigenetic regulators (e.g., TET2 and KMT2D), may play a role in the development of iTCL-GI, particularly in the CD4+ and certain CD8+ subpopulations (22, 23). In this case, the JAK2 mutation indicates the possibility of abnormal activation of the JAK-STAT signaling pathway, although this hypothesis requires further verification through additional studies.

Currently, the treatment strategy for iTCL-GI remains ambiguous and lacks standardized guidelines. For our patient, glucocorticoid and immunomodulator (thalidomide) therapy was ineffective, and the disease demonstrated resistance with an inadequate response to chemotherapy. The patient failed to demonstrate improvement following chemotherapy with the CHOP-E regimen, which could be attributed to the low proliferative activity of tumor cells (24). Some studies suggest that iTCL-GI may not represent a typical tumorigenic lesion. However, TCR gene rearrangement and NGS results in our patient showed monoclonal proliferation and mutations in the JAK2 and DDX3X genes. Mutations in the JAK2 gene can lead to abnormal cell proliferation. Mutations in the DDX3X gene, on the other hand, showed reduced RNA deconjugase activity, which consequently reduces cell cycle inhibition and transcriptionally activates the NF-κB and MAPK pathways. In this patient, the potential disease-related mutation site is a 3.1% DDX3X p.L220S mutation, predicted to be somatic. Functional analysis suggests this mutation may affect protein function. Another identified mutation is a 56.9% DDX3X p.M187R variant, which is not documented in existing population databases. Functional analysis predicts this mutation may affect protein function. A 48.9% JAK2 p.I1889T mutation was detected. The COSMIC database indicates this mutation has been identified in hematological malignancies, while population databases show it occurs at a frequency of less than 1%. Functional prediction suggests this mutation may affect protein function; its clinical significance remains unclear. Although iTCL-GI typically has a slow clinical progression, the possibility of transformation into aggressive malignancy cannot be ruled out. Therefore, targeted therapy against tumor cells was considered a viable next therapeutic approach. Venetoclax is a selective Bcl-2 inhibitor that promotes apoptosis of tumor cells by inhibiting the Bcl-2 protein (25). Previous studies have demonstrated efficacy with venetoclax in the treatment of relapsed or refractory T-cell acute lymphoblastic leukemia, and it has since been approved for the treatment of CLL and acute myeloid leukemia (AML) (26, 27). Our patient poorly tolerated and was unresponsive to CHOP-E chemotherapy; however, after two months of oral venetoclax administration, peripheral blood counts and body temperature gradually normalized, and the colonic ulcer also improved. This suggests that venetoclax may have potential in treating iTCL-GI. Bcl-2 protein expression is known to be upregulated in patients with iTCL-GI, potentially conferring anti-apoptotic properties to tumor cells and thereby contributing to their prolonged survival. Although iTCL-GI is less reliant on Bcl-2 compared to B-cell lymphoma, therapeutic strategies targeting Bcl-2 could be effective in sustaining disease remission in selected patients. Thus, venetoclax, as an agent targeting Bcl-2, may offer a novel treatment option for this rare type of lymphoma. However, its specific efficacy and applicability require further evaluation, considering individual patient characteristics and pathological features.

This case represents the first report worldwide about using venetoclax to treat iTCL-GI, achieving good results. Nevertheless, it is necessary to investigate the optimal dose and duration of venetoclax for iTCL-GI, the ideal combination of drugs, and the effective time of venetoclax for iTCL-GI in a large-scale prospective study. Venetoclax is a promising potent drug in the treatment of iTCL-GI. This report could provide clues for the future management of similar cases.

## Data Availability

The original contributions presented in this study are publicly available. These data can be accessed through the NGDC (National Genomics Data Center) website under Project ID: PRJCA017285 (https://ngdc.cncb.ac.cn/search/all?&q=PRJCA017285).
